# A Comparative Analysis of Digital Mammography and Digital Breast Tomosynthesis in Japanese Women

**DOI:** 10.7759/cureus.87231

**Published:** 2025-07-03

**Authors:** Akemi Hara, Akihiko Ozaki, Masahiro Wada, Kenji Gonda, Akifumi Hagiwara, Megumi Shirato, Nanami Kariya, Ayu Yasui, Rui Omori, Mika Nashimoto, Toyoaki Sawano, Tomohiro Kurokawa, Kazunoshin Tachibana, Tohru Ohtake, Hiroaki Shimmura, Takayoshi Uematsu

**Affiliations:** 1 Clinical Research Center, Jyoban Hospital of Tokiwa Foundation, Iwaki, JPN; 2 Breast and Thyroid Center, Jyoban Hospital of Tokiwa Foundation, Iwaki, JPN; 3 Department of Breast Surgery, Utsunomiya Central Clinic, Utsunomiya, JPN; 4 Department of Breast Surgery, Fukushima Medical University School of Medicine, Fukushima, JPN; 5 Department of Radiology, Juntendo University School of Medicine, Bunkyo City, JPN; 6 Department of Radiology, The University of Tokyo, Tokyo, JPN; 7 Department of Radiology, Jyoban Hospital of Tokiwa Foundation, Iwaki, JPN; 8 Department of Breast Surgery, St Luke International Hospital, Chuo City, JPN; 9 Graduate School of Public Health, Teikyo University, Itabashi, JPN; 10 Breast Center, Kameda Medical Center, Chiba, JPN; 11 Department of Surgery, Jyoban Hospital of Tokiwa Foundation, Iwaki, JPN; 12 Department of Urology, Jyoban Hospital of Tokiwa Foundation, Iwaki, JPN; 13 Department of Breast Imaging and Breast Interventional Radiology, Shizuoka Cancer Center Hospital, Shizuoka, JPN; 14 Department of Clinical Physiology, Shizuoka Cancer Center Hospital, Shizuoka, JPN

**Keywords:** breast cancer, digital breast tomosynthesis, digital mammography, roc analysis, screening

## Abstract

Introduction

Digital breast tomosynthesis (DBT) addresses the limitations of digital mammography (DM). While DM+DBT is gradually replacing DM in the United States and other Western countries, its adoption in Japan has been limited. This study assessed and compared the diagnostic performance of DM and DM+DBT, with a novel focus on breast density, reader experience, and workflow efficiency in Japanese women who underwent examinations during our early experience shortly after the introduction of DBT.

Methods

A retrospective review of 48 patients (19 with breast cancer and 29 with benign lesions) who underwent both DM and DBT at Jyoban Hospital was conducted. Five readers (two physicians, one medical resident, and two radiologic technologists) assessed the images. Diagnostic accuracy was evaluated using receiver operating characteristic (ROC) curve analysis for breast density and reader experience in both screening and clinical settings. Inter-reader agreement and reading times were assessed, with paired t-tests used to analyze differences in reading times.

Results

DM+DBT achieved higher inter-reader agreement than DM (Kappa: 0.708, 95% CI: 0.567-0.849 vs. 0.661, 95% CI: 0.511-0.811), though this difference was not statistically significant (p = 0.653). In screening settings, DM+DBT significantly improved the area under the curve (AUC; 0.750, 95% CI: 0.717-0.784 vs. 0.709, 95% CI: 0.675-0.743; p = 0.005); in clinical settings, the increase was not significant (0.878, 95% CI: 0.834-0.921 vs. 0.844, 95% CI: 0.797-0.891; p = 0.153). For dense breasts, AUC improved notably with DBT (screening: 0.766, 95% CI: 0.699-0.833 vs. 0.684, 95% CI: 0.612-0.756; p = 0.007; clinical: 0.936, 95% CI: 0.883-0.990 vs. 0.827, 95% CI: 0.740-0.914; p = 0.023), but no significant differences were observed in non-dense breasts. All readers improved with DBT, except the medical resident. Reading times increased significantly, from 32.5-82.5 seconds (DM) to 71.3-113.8 seconds (DM+DBT) (p = 0.006).

Conclusion

DM+DBT improves diagnostic accuracy, particularly for dense breasts in Japanese women, but longer reading times and reader experience may limit its widespread adoption.

## Introduction

Breast cancer is the most common cancer among women, with 2.3 million new cases reported and ranking as the leading cause of cancer-related deaths in 2020, according to the WHO [1]. Early detection significantly improves outcomes [2,3]. In Japan, breast cancer is also the most common cancer among women, with 92,153 new cases reported in 2020 [4,5].

Digital mammography (DM), the standard technology for breast cancer screening, demonstrates high sensitivity and specificity, particularly in patients with non-dense breasts [3-6]. However, its performance varies by breast density. In patients with dense breasts, overlapping tissue reduces lesion visibility [6], making the diagnostic limitations of DM more pronounced [3,7-8]. Consequently, screening mammography is considered less suitable for Japanese women, who often have dense breasts [9-12].

Digital breast tomosynthesis (DBT), developed to address the limitations of DM, has gained attention for its ability to reduce tissue overlap through multiple tomographic images. This is particularly beneficial for enhancing lesion detection in patients with dense breasts [7,13-15]. A systematic review published in 2021 revealed that adding DBT to DM increases both cancer detection rates and positive predictive value (PPV) [16]. Additionally, a 2024 systematic review evaluated the effectiveness of tomosynthesis in individuals with dense breasts and those at higher risk [17]. Screening with DBT, compared to DM alone, has also been associated with a lower risk of advanced breast cancer among women with extremely dense breasts and at high risk of developing breast cancer [18].

Despite these benefits, DBT is only weakly recommended in the 2022 breast-imaging guidelines in Japan [14-17,19]. While these guidelines acknowledge improved sensitivity and specificity, based primarily on Western cohorts, they also highlight practical challenges such as longer reading times, difficulty in differentiating postoperative changes, and limited evidence for follow-up imaging [7,14]. Notably, none of the referenced trials included Japanese women, whose breast density profile differs markedly from Western populations (dense breasts ≈60-70% in Japan vs. ≈40% in Europe/United States) [9]. Since dense tissue directly affects lesion conspicuity, extrapolating Western DBT evidence to Japanese women without local validation may either overestimate or underestimate its clinical value.

To date, only three Japanese studies have examined DBT: a 2018 single-center trial showing better sensitivity and specificity of DM+DBT over DM alone [19]; a 2023 comparison of DM+DBT with breast PET and ultrasound [20]; and a 2021 study evaluating DM+DBT for neoadjuvant response prediction [16]. None of these studies systematically explored the interaction of breast density, reader experience, and workflow efficiency in routine Japanese clinical practice. As a result, DBT has not become widespread in Japan, in contrast to its broader adoption in Western countries.

To address the limited evidence on the effectiveness of DBT in Japanese women, who have higher breast density and remain underrepresented in DBT research, this study was conducted in a real-world clinical setting in Japan. We hypothesized that DM+DBT would significantly improve diagnostic accuracy over DM alone, particularly in Japanese women, due to the high prevalence of dense breast tissue in this population.

The objectives of this study were to: (1) compare the diagnostic accuracy of DM and DM+DBT in detecting breast cancer among Japanese women, with specific attention to breast density and reader experience; (2) assess inter-reader agreement across modalities; and (3) evaluate the impact of adding DBT on reading times and workflow efficiency.

## Materials and methods

Study design and participants

The study included patients who underwent mammography at Jyoban Hospital of Tokiwa Foundation after February 2024. The hospital introduced the AMULET SOPHINITY system (Fujifilm, Tokyo, Japan) on February 14, 2024, and began its operation the following day. By October 31, a total of 1,392 DBT examinations had been performed, including re-examinations for some patients.

This study was conducted as an exploratory, feasibility-focused investigation to evaluate the impact of DM+DBT integration into the clinical workflow and to guide future practice. As such, a formal sample size calculation was not conducted, in line with the study’s primary objective of trend identification rather than hypothesis testing.

From this database, 48 unique patients were retrospectively selected using stratified purposive sampling. The primary inclusion criteria were: (1) availability of both DM and DBT images; (2) confirmed diagnosis via histopathology or follow-up imaging; and (3) a balanced distribution of breast density categories and lesion types. The final cohort consisted of 19 malignant, 23 benign, and 6 normal cases. All non-malignant cases (n = 29) were confirmed through histological and imaging analyses, including ultrasound. Stratification was manually applied to ensure balanced representation of both dense and non-dense breasts, as well as malignant, benign, and normal lesions, based on prior diagnostic reports.

Breast parenchymal density, which affects imaging interpretation, was recorded as a dichotomous variable based on diagnostic reports. Breast density was classified using J-RADS, an adaptation of Breast Imaging Reporting and Data System (BI-RADS), into dense (heterogeneous/extremely dense) and non-dense (scattered fibroglandular/predominantly fatty) categories [3,14]. All density evaluations were derived from the diagnostic database to ensure consistency. The start and completion times of image interpretation were also recorded to assess reading duration.

Image acquisition

All patients underwent two-view DM followed by additional DBT imaging using the same equipment, ensuring consistent breast compression and positioning. The examinations were performed using a high-resolution, narrow-angle DBT system (Mammary; AMULET SOPHINITY, Fujifilm, Tokyo, Japan), which utilized a 20-degree tube motion. While the use of a single-vendor system may limit generalizability, AMULET SOPHINITY is widely adopted in Japanese clinical settings, making the findings relevant for domestic practice.

Readers

In Japan, it is common for breast physicians with official screening mammography certification to interpret mammograms. Radiologic technologists are also permitted by government regulation to assist with interpretation (though not diagnosis).

The study included five readers with varying experience levels: two breast physicians (20 and 18 years of mammography reading experience, including 2 and 1 years of tomosynthesis experience), one medical resident (1 year of mammography reading experience), and two radiologic technologists (20 and 7 years of mammography reading experience, both with one year of tomosynthesis experience). All readers, except the resident, regularly participated in clinical image interpretation.

No specific training or calibration session was conducted prior to this study. This decision was intentional, to simulate real-world variability and to examine how reader performance is influenced by prior clinical experience, particularly with limited exposure to DBT.

Reading method

The image reading took place at Jyoban Hospital of the Tokiwa Foundation on November 25-26, 2024. The examiner was present next to the readers, and questions were randomly presented from the dataset. The study aimed to measure the reading time for both DM alone and DM combined with DBT; therefore, readers were prompted to rate each case first with the two-dimensional image alone, followed by DBT.

To minimize recall bias, all assessments were conducted under blinded conditions. Readers were unaware of final diagnoses and were not informed of their own or others’ prior responses. All image readings were conducted independently in a controlled radiology reading room using diagnostic-grade 5-megapixel monitors under standard ambient lighting. No image enhancement or decision support tools were used during interpretation.

Each breast image was assigned a score by the reviewers based on the BI-RADS classification, ranging from 1 (normal) to 5 (highly suggestive of malignancy). The scores were interpreted as follows: 1 indicating negative with no abnormality, 2 for benign findings (considered either positive or negative), 3 for probably benign (positive), 4 for suspicious of malignancy (positive), and 5 for highly suggestive of malignancy (positive).

Outcomes

In this study, two distinct outcome measures were established based on the BI-RADS scoring system to evaluate diagnostic performance in different clinical contexts.

Screening Setting

This outcome aimed to identify lesions requiring further evaluation, including both malignant and benign findings. Positive cases encompassed all lesions classified as BI-RADS 3 or higher, while negative cases were limited to normal findings (BI-RADS 1 or 2).

Clinical Setting

This outcome focused on differentiating malignant lesions from benign lesions and normal findings. Positive cases were defined as those with pathologically or clinically confirmed malignant lesions (BI-RADS 4 or higher), while negative cases included both benign lesions (BI-RADS 2 or 3) and normal findings (BI-RADS 1).

The reference standard, designated as "Actual Label" in our database, was determined through pathological confirmation or definitive clinical diagnosis and categorized into three classifications: normal findings (absence of suspicious lesions), benign lesions (confirmed non-malignant abnormalities), and malignant lesions (pathologically confirmed cancer). Final reference labels were assigned based on pathology reports or consensus clinical diagnosis by attending breast specialists, ensuring consistency and clinical relevance.

Data analysis

First, inter-reader agreement was assessed using Light’s kappa coefficients, with agreement interpreted according to Landis and Koch’s criteria. This method was used to evaluate the overall consistency among the five readers when interpreting BI-RADS scores. To compare the kappa values between DM and DM+DBT, a Z-test was performed based on their respective standard errors. Agreement levels were interpreted using Landis and Koch’s classification, with substantial agreement (0.61-0.80) and almost perfect agreement (0.81-1.00).

Second, diagnostic performance was evaluated using two outcome measures. For the screening setting, sensitivity was calculated at BI-RADS ≥3 and specificity at BI-RADS ≤2. For the clinical setting, sensitivity was calculated at BI-RADS ≥4 and specificity at BI-RADS ≤3. This classification allowed us to distinguish between early lesion detection (screening) and accurate diagnosis of malignancy (clinical), following standard clinical thresholds.

Statistical analysis included four components. First, McNemar’s tests compared sensitivity and specificity between DM and DM+DBT to examine whether the addition of DBT led to statistically significant changes in diagnostic classification within the same patients. ROC curve analysis with AUC calculations used DeLong’s test to evaluate significant differences, providing a robust non-parametric method for comparing overall diagnostic accuracy between modalities. Second, stratified analyses by breast density (dense breasts vs. non-dense breasts) included ROC analyses for each category in both the screening and clinical settings, in order to assess whether breast density modified the diagnostic benefit of DBT, a relevant factor in Japanese populations with higher breast density prevalence.

Third, individual reader performance was evaluated using ROC analysis to explore whether the diagnostic impact of DBT varied by reader experience or professional background. Finally, interpretation times were analyzed to assess workflow implications, with a paired t-test used to evaluate the statistical significance of differences in reading times between DM and DM+DBT. This comparison quantified the practical time cost of using DBT in real-world reading scenarios. All analyses were performed using R Studio (version 4.4.1), with statistical significance set at p < 0.05.

## Results

For inter-reader agreement, the overall Light’s kappa for DM and DM+DBT was 0.661 (95% CI: 0.511-0.811) and 0.708 (95% CI: 0.567-0.849), respectively. A Z test comparing these values yielded Z = -0.45 (p = 0.653), indicating that the difference was not statistically significant.

For diagnostic performance, DM+DBT showed improved sensitivity (0.54 ± 0.10 vs. 0.46 ± 0.07), identical specificity (0.93 ± 0.04), and slightly better accuracy (0.69 ± 0.05 vs. 0.65 ± 0.03) in the screening setting. In the clinical setting, DM+DBT demonstrated higher sensitivity (0.67 ± 0.21 vs. 0.51 ± 0.15), while specificity (0.96 ± 0.00 vs. 0.97 ± 0.00) and accuracy (0.90 ± 0.04 vs. 0.88 ± 0.02) were comparable (Table 1).

**Table 1:** Diagnostic performance metrics of digital mammography and digital breast tomosynthesis by outcome measures (mean ± SD). DM: Digital Mammography; DBT: Digital Breast Tomosynthesis.

Outcome	Metric	DM (Mean ± SD)	DM+DBT (Mean ± SD)
Screening setting	Sensitivity (≥ 3)	0.46 ± 0.07	0.54 ± 0.10
	Specificity (≤ 2)	0.93 ± 0.04	0.93 ± 0.04
	Accuracy (≥ 3)	0.65 ± 0.03	0.69 ± 0.05
Clinical setting	Sensitivity (≥ 4)	0.51 ± 0.15	0.67 ± 0.21
	Specificity (≤ 3)	0.97 ± 0.00	0.96 ± 0.00
	Accuracy (≥ 4)	0.88 ± 0.02	0.90 ± 0.04

**Figure 1:**
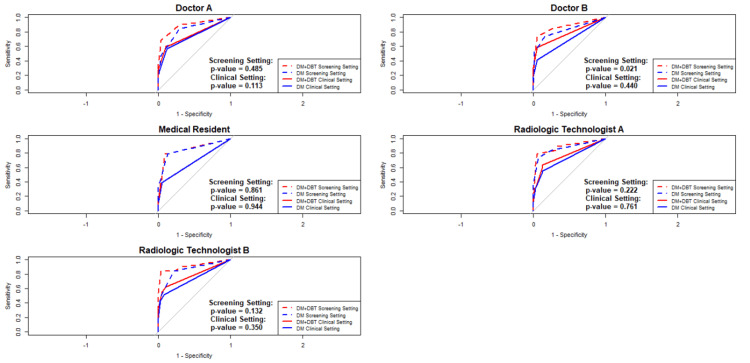
Reader-specific performance analysis of digital mammography and digital breast tomosynthesis using receiver operating characteristic curves†. † In the ROC curve for the medical resident, the dashed lines representing the DM+DBT clinical setting and the DM clinical setting are completely overlapping. DM: Digital Mammography; DBT: Digital Breast Tomosynthesis; ROC: Receiver operating characteristic.

While four of the five readers demonstrated improved AUCs with DM+DBT, statistically significant differences were seen only in the screening setting with doctor B. The medical resident showed no observable benefit (screening setting: p = 0.861; clinical setting: p = 0.944) (Figure 1). This pattern suggests that the impact of DBT may vary depending on reader experience, with experienced readers demonstrating more consistent diagnostic gains.

**Figure 2:**
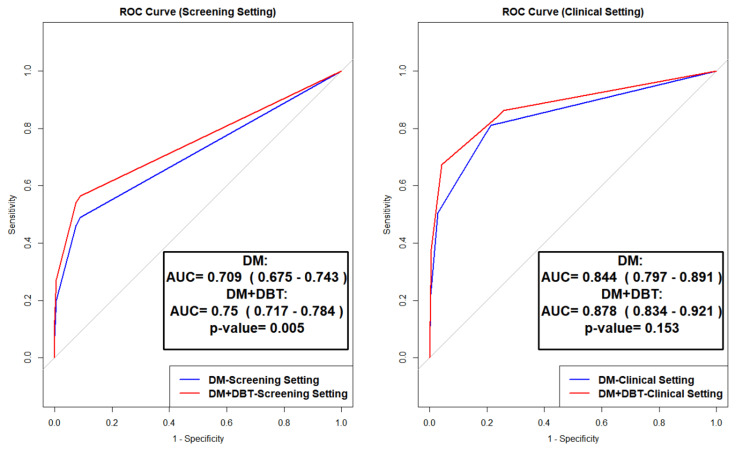
Comparative diagnostic performance of digital mammography versus digital breast tomosynthesis using receiver operating characteristic curves. ROC: Receiver operating characteristic; DM: Digital Mammography; DBT: Digital Breast Tomosynthesis.

Figure 2 shows the ROC curve analyses comparing DM and DM+DBT in screening and clinical settings. In the screening setting, DM+DBT demonstrated significantly better performance than DM alone (AUC: 0.750, 95% CI: 0.717-0.784 vs. AUC: 0.709, 95% CI: 0.675-0.743; p = 0.005). In the clinical setting, although DM+DBT yielded a higher AUC (0.878, 95% CI: 0.834-0.921) than DM (0.844, 95% CI: 0.797-0.891), this difference was not statistically significant (p = 0.153), and the confidence intervals notably overlap.

**Figure 3:**
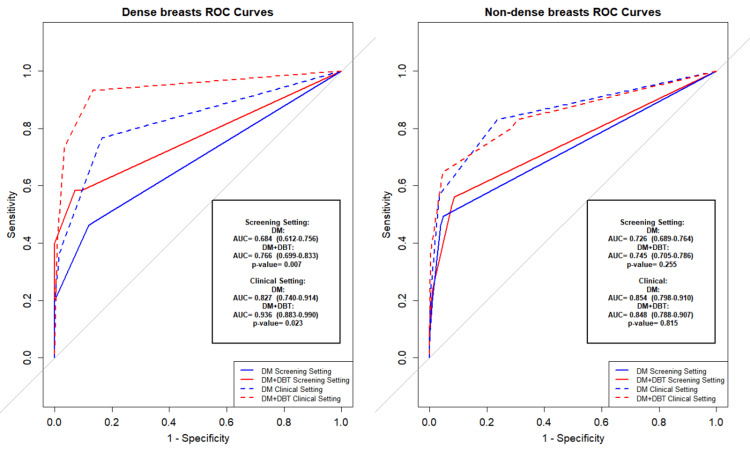
Impact of breast density on diagnostic performance: receiver operating characteristic analysis of digital mammography versus digital breast tomosynthesis. ROC: Receiver operating characteristic; DM: Digital Mammography; DBT: Digital Breast Tomosynthesis.

Figure 3 presents ROC curves stratified by breast density. In dense breasts, DM+DBT significantly outperformed DM in both the screening (AUC: 0.766, 95% CI: 0.699-0.833 vs. 0.684, 95% CI: 0.612-0.756; p = 0.007) and clinical settings (AUC: 0.936, 95% CI: 0.883-0.990 vs. 0.827, 95% CI: 0.740-0.914; p = 0.023). However, in non-dense breasts, no significant differences were observed. In the screening setting, the AUCs were 0.745 (95% CI: 0.705-0.786) for DM+DBT vs. 0.726 (95% CI: 0.689-0.764) for DM (p = 0.255); in the clinical setting, AUCs were 0.848 (95% CI: 0.788-0.907) for DM+DBT vs. 0.854 (95% CI: 0.798-0.910) for DM (p = 0.815). These overlapping confidence intervals and non-significant p-values suggest no meaningful performance difference in non-dense breasts.

For reading time, DM reading times ranged from 32.5 to 82.5 seconds per case, with experienced readers completing cases more quickly. The addition of DBT increased reading times by 1.5- to 2-fold, ranging from 71.3 to 113.8 seconds, while maintaining similar efficiency patterns across readers. A paired t-test confirmed that the increase in reading times with DBT was statistically significant (p = 0.006) (Appendix 1).

## Discussion

Our analysis revealed several important insights into the diagnostic value of DM and DM+DBT for breast cancer detection. ROC curve analysis demonstrated the superior performance of DBT in dense breasts (AUC: 0.936 vs. 0.827, p = 0.023), while no added benefit was observed in non-dense breasts (AUC: 0.848 vs. 0.854, p = 0.815). These results align with previous reports such as Ohashi R et al., though earlier studies often did not stratify by breast density [20]. Given the limited incremental benefit in non-dense breasts, selective use of DBT may help conserve clinical resources without compromising diagnostic accuracy.

Regarding inter-reader agreement, a numerical improvement with DBT was observed (Light’s kappa: 0.708 vs. 0.661), although this was not statistically significant (p = 0.653). Diagnostic performance gains were more evident among experienced readers, for example, Doctor B showed significant improvement in the screening setting (p = 0.021), while the medical resident showed minimal benefit. This suggests that sufficient prior experience with DM is essential for the effective use of DBT. The longer average reading time for DBT (41.5 seconds per case) and limited benefit among novice readers highlight the need for structured reader training. Prior studies have shown that targeted DBT training, including case-based tutorials and calibration exercises, can significantly enhance performance, particularly for less experienced readers [21].

We also observed a trade-off between sensitivity and specificity: DBT improved sensitivity but tended to reduce specificity. This may be partly due to the use of DM-based interpretation frameworks for DBT, as well as the relative inexperience of some readers. With accumulated experience and dedicated DBT interpretation criteria, specificity may improve alongside sensitivity. Standardized training protocols and diagnostic guidelines could help mitigate this issue.

Finally, longer interpretation times pose a barrier to DM+DBT integration in high-volume settings. Synthesized mammography, which reconstructs 2D images from DBT data, may reduce reading time while maintaining diagnostic accuracy. Recent studies suggest that newly developed synthesized mammograms, generated as a form of artificial intelligence-supported DBT reading method, offer performance comparable to conventional DM+DBT [22-24], and their use could streamline workflows. Further studies are needed to evaluate their effectiveness in screening contexts worldwide.

This study has several limitations. First, the single-institution design with 48 patients and five readers limits generalizability due to sample size, demographics, and institutional factors. As all cases were drawn from one site, local protocols, equipment use, and patient characteristics may not reflect broader clinical settings. In particular, the findings were derived using a single DBT system (AMULET SOPHINITY), and diagnostic performance may vary across different vendors or system settings. Second, the retrospective design introduces selection and verification bias, as all cases were confirmed histopathologically. The inclusion of known diagnoses may overestimate diagnostic performance compared to real-world screening, and reader familiarity with the dataset could have influenced interpretations. These biases may have inflated sensitivity estimates by reducing diagnostic uncertainty and thus may not reflect typical screening conditions. Future prospective studies using blinded, sequential case review are needed to mitigate this effect. Third, key clinical outcomes such as recall rates and downstream healthcare utilization were not evaluated. Finally, the short duration following DBT implementation also limited the ability to assess long-term reader adaptation. The lack of improvement among less experienced readers suggests that DBT may not benefit all users equally. This highlights the need for structured training and reader calibration prior to broader implementation. Future studies should evaluate whether standardized DBT interpretation protocols improve inter-reader consistency, particularly among novice readers, and should consider multicenter designs to enhance external validity. In addition, workflow strategies such as synthesized mammography and AI-assisted image triage may help offset the time burden introduced by DBT.

However, this is the first multi-observer performance study to meticulously examine the accuracy and benefits of both DM and DBT in Japan, particularly focusing on variables such as breast density and readers' experience. The research also explores their influence on reading times and diagnostic outcomes.

## Conclusions

In conclusion, the use of DM+DBT enhanced diagnostic accuracy for breast cancer detection and diagnosis, particularly in dense breasts among Japanese women. While DM+DBT improved diagnostic performance, especially among experienced readers, longer interpretation times and limited improvement among novice readers underscore the need for targeted training. To support effective clinical adoption, structured reader education and experience-based calibration are recommended prior to implementation. Future research should explore the role of AI-assisted interpretation and synthesized mammography in reducing workflow burden while maintaining diagnostic accuracy, especially in high-volume screening settings. Larger, multicenter prospective studies in Japanese populations are warranted to validate these findings and guide evidence-based integration into routine practice.

## Appendices

Appendix 1

**Table 2:** Reading time analysis of digital mammography and digital breast tomosynthesis by reader experience level. DM: Digital Mammography; DBT: Digital Breast Tomosynthesis.

Evaluator	Modality	Reading time per case (seconds)
Doctor A	DM	51.2
	DM+DBT	113.8
Doctor B	DM	32.5
	DM+DBT	90
Medical Resident	DM	56.3
	DM+DBT	87.5
Radiologic Technologist A	DM	82.5
	DM+DBT	110
Radiologic Technologist B	DM	42.5
	DM+DBT	71.3
